# Characterization of Plasma Protein Alterations in Pregnant and Postpartum Individuals Living With HIV to Support Physiologically-Based Pharmacokinetic Model Development

**DOI:** 10.3389/fped.2021.721059

**Published:** 2021-10-13

**Authors:** Sherry Zhao, Mary Gockenbach, Manuela Grimstein, Hari Cheryl Sachs, Mark Mirochnick, Kimberly Struble, Yodit Belew, Jian Wang, Edmund V. Capparelli, Brookie M. Best, Tamara Johnson, Jeremiah D. Momper, Anil R. Maharaj

**Affiliations:** ^1^Division of Pediatrics and Maternal Health, Office of Rare Diseases, Pediatrics, Urologic and Reproductive Medicine, Office of New Drugs, Center for Drug Evaluation and Research, U.S. Food and Drug Administration, Silver Spring, MD, United States; ^2^Office of Clinical Pharmacology, Office of Translational Sciences, Center for Drug Evaluation and Research, U.S. Food and Drug Administration, Silver Spring, MD, United States; ^3^Boston University School of Medicine, Boston, MA, United States; ^4^Division of Antivirals, Office of Antimicrobials, Office of New Drugs, Center for Drug Evaluation and Research, U.S. Food and Drug Administration, Silver Spring, MD, United States; ^5^Office of New Drugs, Center for Drug Evaluation and Research, U.S. Food and Drug Administration, Silver Spring, MD, United States; ^6^Skaggs School of Pharmacy and Pharmaceutical Sciences, University of California, San Diego, San Diego, CA, United States; ^7^Pediatrics Department, School of Medicine, San Diego-Rady Children's Hospital San Diego, University of California, San Diego, San Diego, CA, United States; ^8^Faculty of Pharmaceutical Sciences, The University of British Columbia, Vancouver, BC, Canada

**Keywords:** albumin, α1-acid glycoprotein, pregnancy, postpartum, HIV, PBPK

## Abstract

**Background:** Alterations in plasma protein concentrations in pregnant and postpartum individuals can influence antiretroviral (ARV) pharmacokinetics. Physiologically-based pharmacokinetic (PBPK) models can serve to inform drug dosing decisions in understudied populations. However, development of such models requires quantitative physiological information (e.g., changes in plasma protein concentration) from the population of interest.

**Objective:** To quantitatively describe the time-course of albumin and α1-acid glycoprotein (AAG) concentrations in pregnant and postpartum women living with HIV.

**Methods:** Serum and plasma protein concentrations procured from the International Maternal Pediatric Adolescent AIDS Clinical Trial Protocol 1026s (P1026s) were analyzed using a generalized additive modeling approach. Separate non-parametric smoothing splines were fit to albumin and AAG concentrations as functions of gestational age or postpartum duration.

**Results:** The analysis included 871 and 757 serum albumin concentrations collected from 380 pregnant (~20 to 42 wks gestation) and 354 postpartum (0 to 46 wks postpartum) women, respectively. Thirty-six and 32 plasma AAG concentrations from 31 pregnant (~24 to 38 wks gestation) and 30 postpartum women (~2–13 wks postpartum), respectively, were available for analysis. Estimated mean albumin concentrations remained stable from 20 wks gestation to term (33.4 to 34.3 g/L); whereas, concentrations rapidly increased postpartum until stabilizing at ~42.3 g/L 15 wk after delivery. Estimated AAG concentrations slightly decreased from 24 wks gestation to term (53.6 and 44.9 mg/dL) while postpartum levels were elevated at two wks after delivery (126.1 mg/dL) and subsequently declined thereafter. Computational functions were developed to quantitatively communicate study results in a form that can be readily utilized for PBPK model development.

**Conclusion:** By characterizing the trajectory of plasma protein concentrations in pregnant and postpartum women living with HIV, our analysis can increase confidence in PBPK model predictions for HIV antiretrovirals and better inform drug dosing decisions in this understudied population.

## Introduction

The Panel on Treatment of Pregnant Women with HIV Infection and Prevention of Perinatal Transmission recommends that all pregnant individuals^1^ living with HIV initiate or maintain antiretroviral (ARV) therapy throughout pregnancy regardless of plasma HIV RNA or CD4 count (1). During the course of pregnancy, a myriad of anatomical and physiological changes occur that can lead to substantial alterations in ARV drug disposition (2, 3). Correspondingly, use of standard adult dosages in pregnant individuals may result in inappropriate drug exposures. Ensuring optimal ARV therapy in pregnant individuals living with HIV confers several benefits including preventing toxicity to both the mother and fetus, decreasing the risk of the development of drug resistance, maintaining viral suppression during pregnancy, and preventing mother-to-child HIV transmission (4). Nonetheless, many drugs have yet to be adequately studied in this population.

Physiologically based pharmacokinetic (PBPK) modeling is an approach that has the potential to provide *a priori* predictions of drug disposition in understudied populations, such as pregnant individuals. By incorporating biologically relevant physiological parameters, models may inform the design of clinical trials in pregnancy and provide estimates on when and to what extent dose adjustments might be needed (5, 6). Owing to the distinct anatomical and physiological differences between pregnant and non-pregnant individuals, several research groups have developed population PBPK models for pregnant individuals (7, 8). Notably, physiological information used to inform such models are predominately derived from Caucasian individuals with low-risk pregnancies. In comparison, pregnant individuals living with HIV represent a demographically diverse population (9). Such individuals typically receive multiple concomitant medications (10). Yet, quantitative physiological information on parameters influencing drug disposition in pregnant individuals living with HIV is limited. Consequently, currently published population PBPK models may not fully reflect the underlying physiology of pregnant individuals living with HIV.

Differences in plasma protein concentrations between individuals can lead to pronounced changes in fractions of unbound drug in plasma and subsequent alterations in systemic drug disposition, particularly for drugs that exhibit high degrees of plasma protein binding (>90% protein binding) (11, 12). Albumin and α1-acid glycoprotein (AAG) represent the two major plasma proteins responsible for binding of a majority of exogenously administered compounds (13). Literature reports evaluating gestational changes in plasma protein concentrations in pregnant individuals have been well-documented in the literature (14–16). However, due to a lack of information specific to pregnant individuals living with HIV, it remains unclear if historical data derived from individuals without HIV infection can be used to inform the development of PBPK models for this population.

After delivery, many of the physiologic changes associated with pregnancy take weeks to months to revert to their pre-pregnancy baseline (17). Studies on antibiotic pharmacology have demonstrated marked alterations in drug pharmacokinetics (PK) between early (2–3 days post-delivery) and late (>4 months post-delivery) postpartum periods (18, 19). Yet, few PBPK models have specifically been developed to estimate drug PK during this unique physiologic period (17). To inform the development of PBPK models capable of informing ARV dosing decisions in postpartum individuals living with HIV, data are needed to describe the physiological transition following delivery back to a non-pregnant state. Considering the important influence that plasma protein binding exerts on systemic drug disposition, this study sought to (1) quantitatively describe the time-course of serum/plasma protein concentrations (i.e., albumin and AAG) in pregnant and postpartum women living with HIV, (2) compare concentrations to values reported in pregnant and postpartum women without HIV infection, and (3) generate computational functions that describe the trajectory of serum/plasma protein concentrations in pregnant and postpartum women living with HIV to inform the prospective development of PBPK models.

## Materials and Methods

### Data Source

Our analysis evaluated serum and plasma protein concentrations procured from the International Maternal Pediatric Adolescent AIDS Clinical Trial (IMPAACT) Protocol 1026s (P1026s), a multicenter, multi-arm, open-label prospective opportunistic study of ARV and tuberculosis medications in pregnant and postpartum women (20). Pregnant women living with HIV (PWLH) as well as postpartum women receiving ARV medications as specified by the study protocol were eligible for enrollment. Each participant's ARV regimen was prescribed and managed by their treating physician. Key inclusion criteria during pregnancy included confirmed HIV diagnosis, receiving stable ARV treatment, and ≥20 wks gestation. Exclusion criteria included concomitant use of medications known to interfere with the PK of the medications being evaluated, multiple pregnancy (i.e., carrying multiple fetuses), and clinical or laboratory evidence of drug toxicity that would likely require changes in the medication regimen during the study. The protocol specified that women enrolled during pregnancy would remain in the study up to 24 wks after delivery. Intensive PK sampling and/or laboratory measurements were taken on multiple occasions including in the second trimester (20 to 26 gestational wks), third trimester (30 to 38 gestational wks), at delivery, and during the postpartum period (2 to 24 wks postpartum). Our analysis leveraged serum albumin and plasma AAG concentrations collected as part of these clinical evaluations. Demographic and anthropometric data including maternal age, weight, height, ethnicity, and country were collected. Additionally, pre-pregnancy weight was collected, if available.

### Albumin in Pregnant and Postpartum Women Living With HIV

The time-course of serum albumin concentrations in pregnant and postpartum women living with HIV were described using a generalized additive modeling approach. Separate models were developed for the pregnant and postpartum periods. The start of the postpartum period coincided with the date of delivery. Models were developed using the Generalized Additive Models for Location, Scale, and Shape (gamlss) package in R (version 4.0.3; R Foundation for Statistical Computing, Vienna, Austria) (21). Non-parametric penalized beta-splines were used to model serum albumin concentrations (response variable) as a function of gestational age (weeks) or postpartum duration (weeks) (22). Models incorporating different error-distribution assumptions were generated and compared. In addition, models that permitted for changes in response variable variance as a function of gestational age or postpartum duration were evaluated. Model selection was guided by a combination of visual and quantitative appraisals including goodness-of-fit plots (i.e., quantile-quantile plots) and generalized Akaike information criterion (AIC) values (23). For the selected pregnancy and postpartum models, theoretical (model-based) time-dependent albumin quantiles associated with the 2.5^th^, 10^th^, 25^th^, 50^th^, 75^th^, 90^th^, and 97.5^th^ percentiles were estimated. To evaluate the fit of model-derived quantiles, the percentage of observed data falling below each respective quantile was summarized for each dataset (pregnancy and postpartum). For models exhibiting an appropriate fit to the data, the percentage of observed data corresponding to each theoretical quantile should be congruent (e.g., 2.5% of observed data should fall below the model-derived quantile for the 2.5^th^ percentile).

### AAG in Pregnant and Postpartum Women Living With HIV

An initial assessment of the analysis dataset indicated that plasma AAG concentrations were not consistently reported. For each respective cohort (i.e., pregnancy and postpartum), <40 observations were available. Owing to the disparate nature of available AAG concentrations, the ability to examine different error distributions as well as alterations response variable variance, as conducted above, was limited. As such, our analysis exclusively focused on describing the time-course of the central tendency of plasma AAG concentrations in pregnant and postpartum women. Using the *smooth.spline()* function in R, separate cubic smoothing splines were fit to AAG concentrations (response variable) as functions of gestational age (weeks) or postpartum duration (weeks) in cohorts of pregnant and postpartum women, respectively.

### Comparison to Serum/Plasma Concentrations From Women Without HIV Infection

Estimated serum/plasma protein concentrations (albumin and AAG) derived from our analysis were graphically compared to estimates for pregnant and postpartum women without HIV infection published by researchers affiliated with leading PBPK modeling platforms (i.e., Certara Simcyp™ and Open Systems Pharmacology PK-Sim®). Equations published by Abduljalil et al. and Dallmann et al. (7, 8) were used to facilitate these comparisons for pregnant women. Protein concentration estimates reflective of postpartum women were derived from Dallmann et al.'s published equation (17). The above-described equations were formulated following quantitative assessments of data collected over multiple publications from women without HIV.

### Development of Computational Functions Describing Serum/Plasma Protein Concentrations in Pregnant and Postpartum Women Living With HIV

Several computational functions were developed to communicate the results of our analysis in a form that can be readily integrated into PBPK modeling platforms. Functions were generated using the open-source software R. For serum albumin, where the analysis included specific considerations for the central tendency, variance, and error distribution, four separate computational functions were developed. Developed functions provided estimates of (1) the arithmetic mean of serum albumin concentrations in pregnancy with increasing gestation, (2) quantiles of serum albumin concentrations in pregnancy, (3) the arithmetic mean of serum albumin concentrations in postpartum women with increasing postpartum duration, and (4) quantiles of serum albumin concentrations in postpartum women.

Due to their lower computational complexity, parametric approximations of developed non-parametric models were incorporated into generated functions. Parametric polynomials were used to approximate the relationship between the central tendency (arithmetic mean) of serum albumin concentrations and time (i.e., gestational age or postpartum duration), as previously defined by corresponding non-parametric models. The degree of the polynomial was sequentially increased until the coefficient of determination (R^2^) describing the polynomial's fit to estimates from the non-parametric model was >0.995. Quantile functions computing time-dependent estimates of serum albumin concentrations at specific percentiles were created by integrating the developed polynomials, describing the mean of albumin concentrations, and statistical considerations for the variance and error distribution of albumin values, as previously defined by corresponding non-parametric models. For AAG, where our analysis solely focused on describing the time-course of the central tendency of plasma AAG values, two computational functions were developed to describe arithmetic mean of AAG concentrations in (1) pregnant and (2) postpartum women. These functions were created using parametric polynomial approximations, as described above.

## Results

### Demographics

Our analysis evaluated 871 serum albumin concentrations from 380 PWLH between ~20 to 42 wks gestation (Table 1). For postpartum women living with HIV, 757 samples were evaluated from 354 women between 0 to 46 wks after delivery. In several instances, final study visits (i.e., 24 wks post-delivery) were delayed, resulting in clinical evaluations occurring up to several weeks after the protocol-defined end date. Two serum albumin concentrations present in the original dataset (1 pregnancy and 1 postpartum) that exhibited relatively high values compared to others (>60 g/L) were excluded from the analysis. Both the pregnant and postpartum albumin datasets were comprised of racially diverse cohorts. Black (i.e., Black African and Black or African American) women represented 47.4 and 46.9% of participants in the pregnancy and postpartum datasets, respectively. The majority of women were enrolled from the United States including 84.5 and 85.3% of women in the pregnancy and postpartum albumin datasets, respectively. The rest were enrolled from international sites. Concomitant ARV medications taken by women included in the albumin analysis are denoted in Supplementary Table 1.

**Table 1:** Demographic characteristics of the pregnant and postpartum women providing serum albumin and plasma AAG samples.

**Demographic Parameters**	**Albumin dataset**		**AAG dataset**	
	**Pregnant** **(380 Women)**	**Postpartum** **(354 Women)**	**Pregnant** **(31 Women)**	**Postpartumn** **(30 Women)**
Country, *n* (%)				
Argentina Botswana Brazil South Africa Thailand Uganda United States	3 (0.8) 12 (3.2) 14 (3.7) 2 (0.5) 20 (5.3) 8 (2.1) 321 (84.5)	1 (0.3) 12 (3.4) 12 (3.4) 2 (0.6) 17 (4.8) 8 (2.3) 302 (85.3)	– – – – – – 31 (100%)	– – – – – – 30 (100%)
Race, *n* (%)				
Asian or Pacific Islander Black African Black or African American Indigenous American Other Unknown/Unavailable White (Caucasian)	24 (6.3) 22 (5.8) 158 (41.6) 3 (0.8) 5 (1.3) 23 (6.1) 145 (38.2)	22 (6.2) 22 (6.2)144 (40.7)3 (0.8)5 (1.4)20 (5.9) 138 (39)	– – 11 (35.5) 1 (3.2) – 8 (25.9) 11 (35.5)	– – 11 (36.7) 1 (3.3) – 8 (26.7) 10 (33.3)
Age (at delivery), years,Median (Q1, Q3)	29 (24, 34)	29 (24, 34)	29 (26.6, 33.5)	30 (27, 33.8)
Weight (pre-pregnancy), kg,Median (Q1, Q3)^1^	67.1 (57.9, 85.4)	67.8 (58.5, 84.8)	69.7 (60.5, 86.2)	69.7 (60.5, 86.2)
Body-Mass Index(pre-pregnancy), kg/m^2^,Median (Q1, Q3)^1^	26.2 (22.7, 32.5)	26.4 (22.9, 32.7)	26.7 (22.8, 31.6)	26.7 (22.8, 31.6)
Weight (at delivery), kg,Median (Q1, Q3)^2^	79.5 (68.3, 94.5)	80.1 (68.5, 94.5)	86.5 (73.4, 97.7)	81.75 (73, 98.2)

*AAG, α1-acid glycoprotein; Q1, First quartile; and Q3, Third quartile. ^1^Pre-pregnancy weights and body mass index values were missing for 116, 107, 8, and 7 individuals from the pregnancy albumin, postpartum albumin, pregnancy AAG, and postpartum AAG datasets, respectively. ^2^Delivery weights were missing for 37, 34, 2, and 2 individuals from the pregnancy albumin, postpartum albumin, pregnancy AAG, and postpartum AAG datasets, respectively*.

Thirty-six plasma AAG concentrations collected from 31 PWLH between ~24 to 38 wks gestation were included in the analysis (Table 1). The analysis additionally included 32 postpartum AAG concentrations collected from 30 women between ~2 to 13 wks post-delivery. Evaluated plasma AAG concentrations were exclusively collected from participants enrolled in the United States. Black (i.e., Black or African American) women comprised 35.5 and 36.7% of the participants included in the pregnancy and postpartum AAG datasets, respectively. All women who contributed AAG concentrations for analysis were taking lopinavir/ritonavir (i.e., Kaletra; Supplementary Table 1).

### Serum Albumin in PWLH

**Figure 1:**
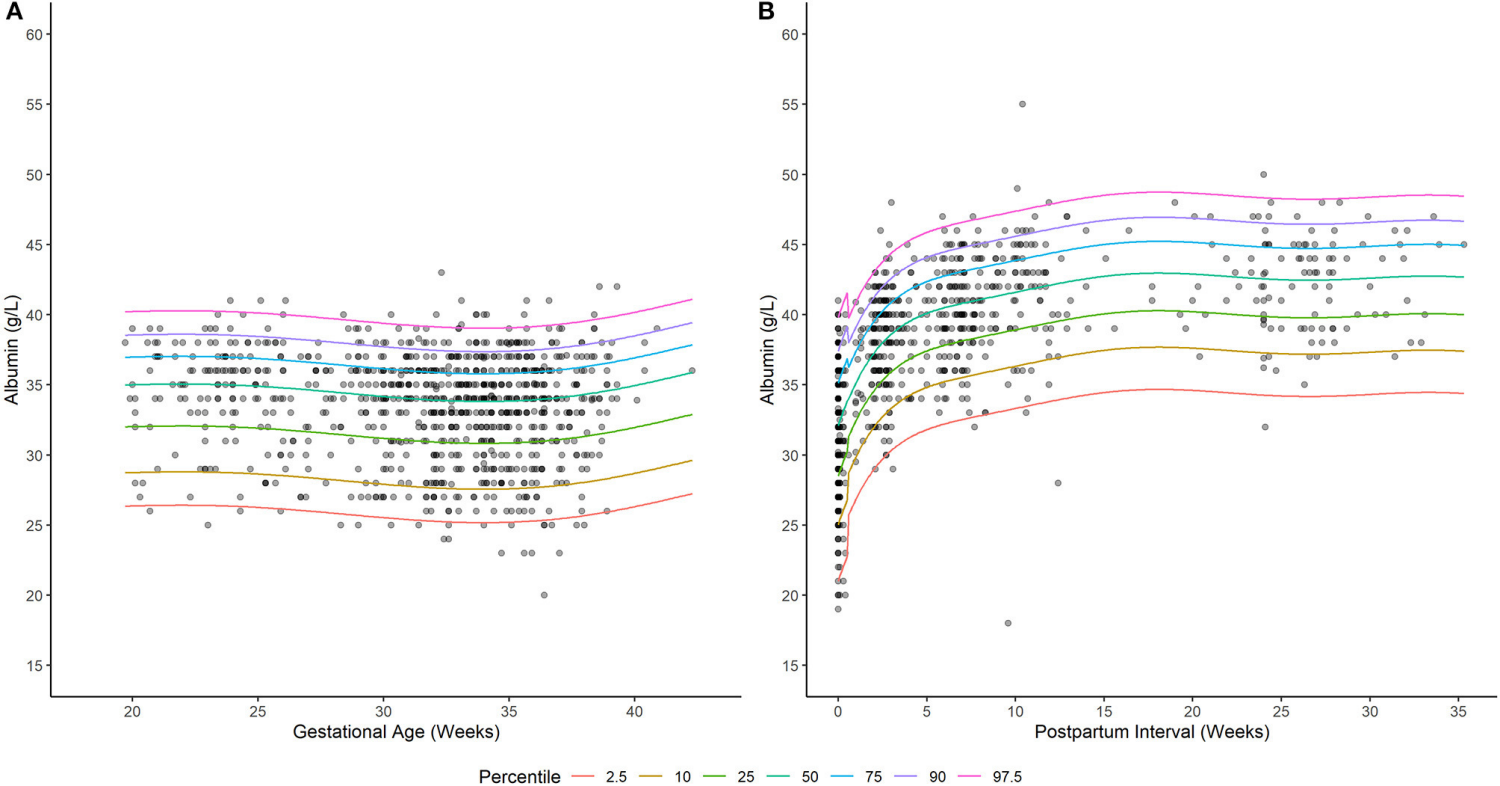
Serum albumin concentrations in **(A)** pregnant and **(B)** postpartum women living with HIV. Solid lines depict model-based quantile estimates at denoted percentiles. Filled-circles depict observed serum albumin concentrations.

A generalized additive model incorporating a non-parametric smoother, a mixture-normal error distribution, and a constant error variance appropriately characterized the relationship between weeks gestation and serum albumin concentrations in PWLH (Supplementary Figure 1; Supplementary Table 2). The selected model utilized a combination of two normal (i.e., gaussian) distributions to describe the spread of serum albumin concentrations. Although an alternative model that additionally permitted for alterations in the variance of albumin concentrations with increasing gestation exhibited a lower AIC value compared to the selected model (ΔAIC = −2.034; Supplementary Table 2), this difference was considered minute and, thus, a constant error variance model was adopted. Model-based quantiles adequately described the time-course of observed albumin concentrations in PWLH beyond 20 wks gestation (Figure 1A). This was additionally demonstrated by the high level of agreement between observed albumin concentrations and model-estimated quantiles (Table 2).

**Table 2:** Percent of observed albumin concentrations in pregnant and postpartum women living with HIV that fall below model-predicted quantiles (associated with specified percentiles).

**Dataset**	**Albumin pregnancy** **dataset**		**Albumin postpartum** **dataset**
**Model**	**P1026s** **(current study)**	**Dallmann et al.** **(8)**	**P1026s** **(current study)**
Predicted Percentile			
2.5% 10% 25% 50% 75% 90% 97.5%	2.64% 9.30% 24.68% 49.71% 73.36% 90.01% 97.82%	1.95% 13.55% 29.97% 69.23% 96.56% 99.66% 100.00%	1.85% 10.2% 25.8% 51.3% 74.4% 89.7% 98.28%

**Figure 2:**
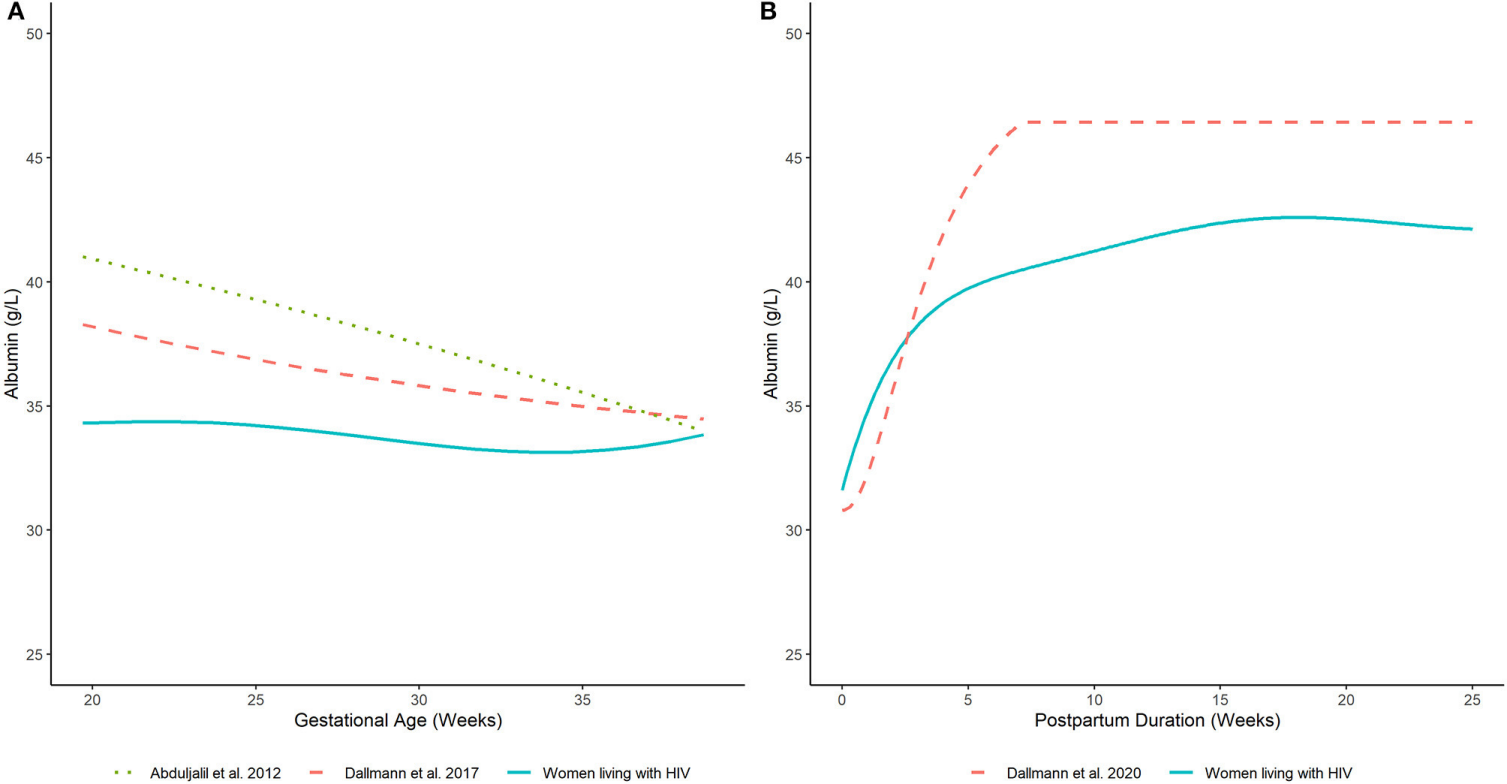
Serum albumin concentrations in **(A)** pregnant and **(B)** postpartum women estimated by our analysis (women living with HIV) and other published equations (women without HIV infection) (7, 8, 17). Lines depict (arithmetic) mean albumin concentration estimates generated by competing models.

The estimated time-course for the central tendency (i.e., arithmetic mean) of serum albumin concentrations is displayed in Figure 2A. Model estimates were corroborated by average values computed from the observed data (Supplementary Table 3). In PWLH, average serum albumin concentrations remained relatively constant from 20 weeks gestations. Estimated values at 20 and 37 wks gestation were similar (34.3 and 33.4 g/L, respectively). In contrast, previously published equations describing the time-course of serum albumin concentrations in pregnant women without HIV infection denoted a decreasing trend (7, 8). For example, estimated serum albumin concentrations based on Dallmann et al.'s publication were 38.2 and 34.7 g/L at 20 and 37 wks gestation, respectively (8). Likewise, estimates from Abduljalil et al.'s published equation were 40.9 and 34.7 g/L, respectively (7). Notably, at term (i.e., 37 wks gestation), estimated values were similar between our analysis and those generated by competing equations.

**Figure 3:**
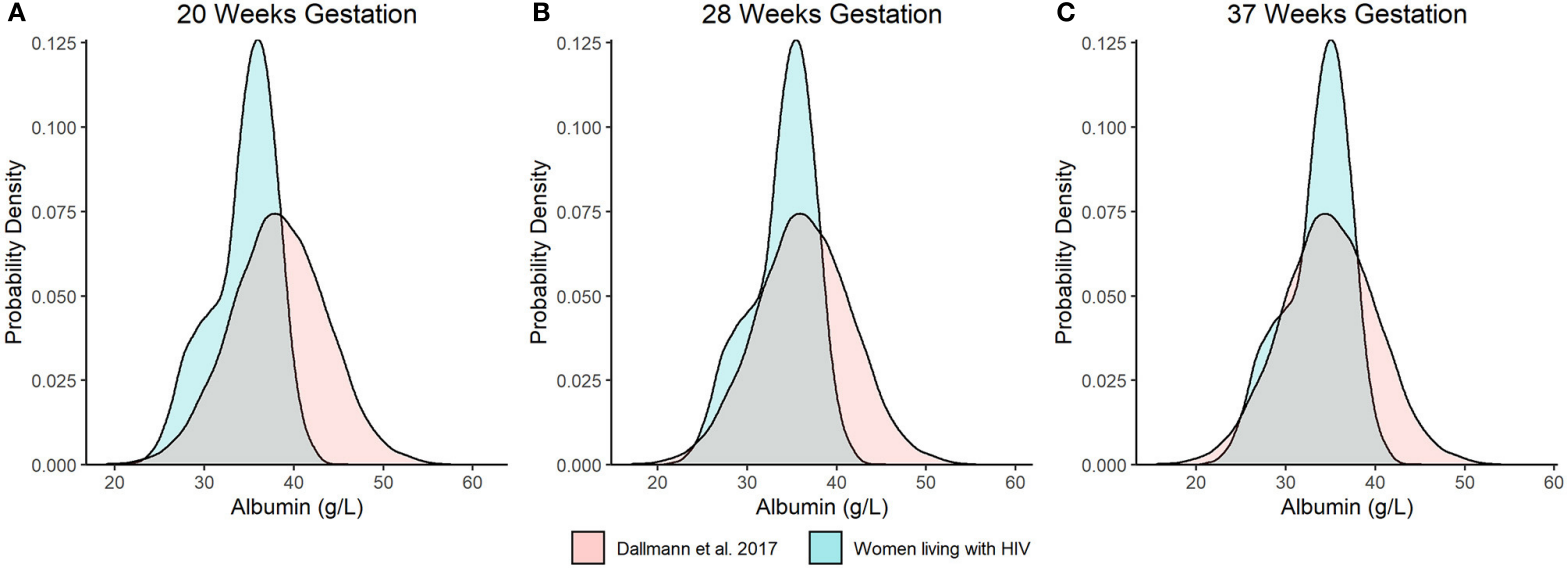
Distributional comparison of predicted serum albumin concentration values at **(A)** 20, **(B)** 28, and **(C)** 37 wks gestation between our model and Dallmann et al.'s published equation (8). Distributions of serum albumin concentrations were generated over 10,000 stochastic simulations from each respective model/equation.

As an additional evaluation, we compared the distribution of model predicted albumin concentrations at 20 wks, 28 wks, and term (i.e., 37 wks gestation) from our analysis to Dallmann et al.'s published equation (Figures 3A–C) (8). For the latter, albumin concentrations were approximated using a normal distribution with a standard deviation of 5.33 g/L. The difference in median values between the two analyses decreased with increasing gestation and were nearly congruent at term, where albumin concentrations of 34.1 and 34.8 g/L were estimated by our analysis and Dallmann et al.'s (8) equation, respectively. However, the distribution of albumin concentrations for PWLH exhibited a slight negative (left) skew whereas estimates from Dallmann et al.'s model were symmetrically distributed. The probability density associated with the upper tails of each respective distribution distinctly differed. At term, Dallmann et al.'s (8) equation exhibited a higher proportion of estimates exceeding 40 g/L compared to our model (Figure 3C). For example, 16.7% of albumin concentrations at term were expected to be >40 g/L based on Dallmann et al.'s (8) model. In comparison, only 1.5% of estimates exceeded this threshold based on our model. An evaluation of the percent of observed data from PWLH that corresponded to quantiles generated from Dallmann et al.'s (8) model further highlighted this distributional mismatch (Table 2). Notably, observed data were discordant with upper model quantiles (75^th^, 90^th^, and 97.5^th^ percentiles).

### Serum Albumin in Postpartum Women Living With HIV

A generalized additive model incorporating a non-parametric smoother, a skew normal (type 2) error distribution, and time-dependent changes in error variance provided an appropriate fit to the data (Supplementary Figure 2; Supplementary Table 4). The selected distribution (i.e., skew normal type 2) exhibited a slight negative (left) skew (24). This is visually depicted in Figure 1B, where differences in albumin concentrations between subsequent lower percentiles are relatively larger compared to upper percentiles. The selected model employed different error variances for albumin concentrations ≤ 0.5 and >0.5 wks postpartum. For ≤ 0.5 wks postpartum, the estimated standard deviation of albumin concentrations was 4.86 g/L; whereas, beyond this period, a standard deviation of 3.64 g/L was estimated. Model quantiles exhibited a suitable fit to the data as demonstrated by the high degree of concordance with observed concentration values (Table 2).

The predicted time-course for the arithmetic mean of serum albumin concentrations during the postpartum period is displayed in Figure 2B. These estimates agreed with average values computed from the observed data (Supplementary Table 5). Average serum albumin concentrations slightly decreased immediately after birth. Estimated albumin concentrations at term (i.e., 37 wks gestation) based on the developed pregnancy model was 33.4 g/L; whereas, a value of 31.6 g/L was estimated at delivery based on the developed postpartum model. Serum albumin concentrations rapidly increased after delivery and stabilized to an average value of ~42.3 g/L after 15 wks postpartum. Estimated albumin values were ~75% (31.6 g/L) of this plateau value at delivery and increased to ~90% (38.3 g/L) by 3 wks postpartum. A similar pattern was depicted by Dallmann et al.'s published equation, which described the trajectory of serum albumin concentrations in postpartum women without HIV infection (17). However, the denoted plateau value, achieved at ~7 wks postpartum, was relatively higher (~46.4 g/L). Nonetheless, the time-course of serum albumin concentrations was relatively similar to our analysis. For example, albumin concentrations estimated by Dallmann et al.'s (17) model were ~66% (30.8 g/L) of their respective plateau value at delivery and increased to ~90% (41.9 g/L) at 4 wks. A distributional comparison between our model and Dallmann et al.'s (17) was not instituted as the latter did not specify the variance and/or distribution of postpartum albumin values.

### AAG in PWLH

**Figure 4:**
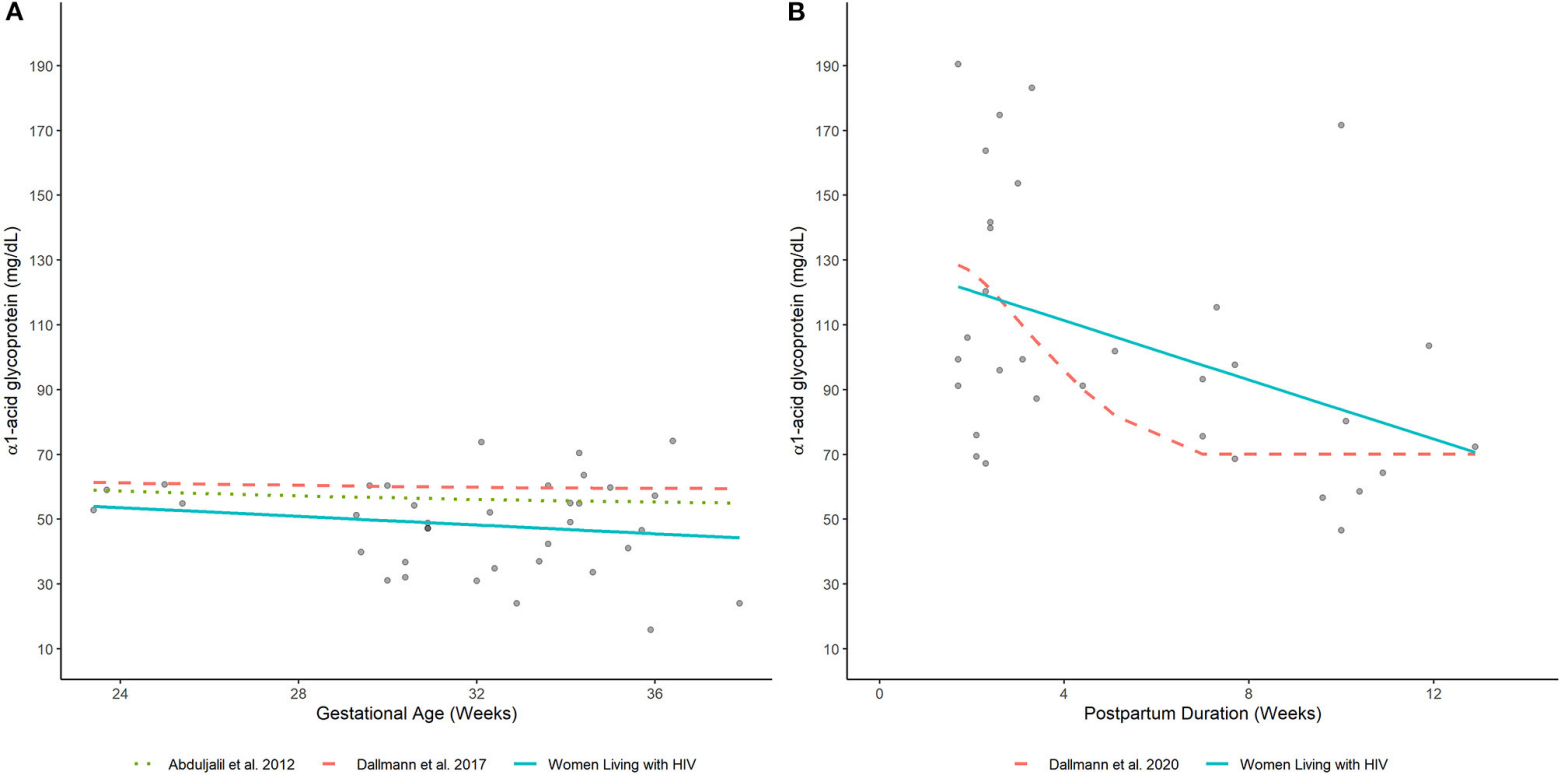
Plasma AAG concentrations in **(A)** pregnant and **(B)** postpartum women estimated by our analysis (women living with HIV) and other published equations (women without HIV infection) (7, 8, 17). Lines depict (arithmetic) mean AAG concentration estimates generated by competing models. Filled-circles depict observed plasma AAG concentrations.

Average plasma AAG concentrations, as estimated by a fitted smoothing spline, decreased linearly between 24- and 37-wks gestation (Figure 4A). Estimated AAG concentrations at these time points were 53.6 and 44.9 mg/dL, respectively. Previously published equations depicting the trajectory of plasma AAG concentrations in women without HIV infection depicted slightly higher values over similar time periods (7, 8). Nonetheless, these equations exhibited similar decreasing trends, albeit with slower rates of decrease. For example, estimated AAG concentrations at 24 and 37 weeks gestation based on Dallmann et al.'s published equation were 61.2 and 59.5 mg/dL, respectively (8); whereas, Abduljalil et al.'s equation depicted concentrations of 58.6 and 55.3 mg/dL, respectively (7).

### AAG in Postpartum Women Living With HIV

Elevated plasma AAG concentrations were observed for samples collected closer to the date of delivery and decreased thereafter (Figure 4B). A smoothing spline fit to the data depicted a linear decreasing trend in average plasma AAG concentrations with increasing time after delivery. The estimated AAG concentration at 2 wks postpartum was 120.4 mg/dL, which was notably higher than the estimated value at term (44.9 mg/dL; 37 wks gestation) determined for PWLH. The fitted trajectory of AAG concentrations did not appear to reach a nadir value and was predicted to be 74.7 mg/dL at 12 wks postpartum. A previously published equation describing AAG concentrations in postpartum women without HIV infection displayed some similarities (17). The AAG concentration at 2 wks postpartum was estimated to be 126.1 mg/dL based on Dallmann et al.'s (17) published equation. Similar to our analysis, AAG concentrations decreased with increasing time after delivery. However, the rate of decline described by Dallmann et al. (17) was notably faster. AAG concentrations rapidly declined until reaching a nadir of 70.1 mg/dL at ~7 wks postpartum.

### Computational Functions

Computational functions that output time-dependent estimates of albumin and AAG concentrations for pregnant and postpartum women living with HIV are provided in the Supplementary Materials. Two types of functions were developed: arithmetic mean and quantile functions. Arithmetic mean functions require one input, either gestational age or postpartum duration (weeks), and output corresponding estimates of serum/plasma protein concentration values. Quantile functions require two inputs: (1) gestational age or postpartum duration (weeks) and (2) a percentile value. These functions output time-dependent estimates of serum albumin concentrations corresponding to specified percentiles. An example script demonstrating the usage of the above-described functions, including how to generate stochastic albumin concentrations for the creation of virtual populations for prospective population PBPK model analyses, has also been provided in the Supplementary Materials.

## Discussion

To our knowledge, this quantitative analysis represents the first to specifically evaluate time-dependent changes in serum/plasma protein concentrations in pregnant and postpartum individuals living with HIV. Our analysis leveraged data collected from the IMPAACT Network's protocol 1026s, representing a large multicenter, multinational protocol designed to evaluate the pharmacokinetics of antiretroviral drugs during and after pregnancy. As such, the current analysis represents the largest assessments of serum/plasma protein concentrations in pregnant and postpartum individuals living with HIV to date. Pregnant individuals are generally excluded from drug development programs and clinical research trials, leading to the current paucity of drug safety and PK information in this population making it challenging to use many licensed drugs safely and effectively (25, 26). Although PBPK model analyses have the potential to generate *a priori* predictions of drug disposition in understudied populations, model development requires anatomical and physiological information specific to target populations of interest. By providing a quantitative description of protein concentrations in pregnant and postpartum individuals living with HIV, our study will serve to improve the development of PBPK models for this population. Furthermore, we have provided computational functions, developed using the open-source software R, to communicate our results in an interactive and transparent manner. Such functions can be directly integrated into PBPK modeling platforms.

Many ARV drugs exhibit high degrees of plasma protein binding (>90%), including several members of the integrase inhibitor and protease inhibitor classes (12, 27, 28). For such drugs, an understanding of the extent of plasma protein binding is considered critical for optimizing ARV therapy. Gestational changes in plasma protein concentrations can impact the extent of ARV plasma protein binding and subsequently contribute to pregnancy-associated alterations in drug PK. For example, the protease inhibitor lopinavir, which displays affinity for both albumin and AAG, exhibits pregnancy-associated changes in protein binding and PK (29). The fraction unbound of lopinavir in plasma increases by ~12–20% in the second and third trimesters of pregnancy compared to postpartum values (30, 31). In addition, apparent clearance of lopinavir (co-administered with ritonavir) is increased by 31–58% between these periods (31–33). The observed increase in fraction unbound aligns with depicted physiological differences in serum/plasma protein concentrations between pregnant and postpartum individuals, as demonstrated by our analysis and along with others (30, 31). However, the observed increase in apparent clearance for lopinavir, which when co-administered with ritonavir exhibits characteristics similar to that of a low extraction ratio drug, exceeds the proportional increase that would be expected if PK alterations were due to changes plasma protein binding alone (34). This indicates that other physiological changes need to be considered in tandem with changes in protein binding to rationally inform ARV dosing in pregnancy. As such, our analysis serves to describe only one facet of the complex physiological changes that occur during pregnancy.

Therapeutic goals of ARV therapy in pregnancy are focused toward maintaining maternal health while preventing vertical viral transmission (4). Correspondingly, both maternal and fetal ARV drug exposures are of interest. Several physiological parameters can influence the extent of fetal drug exposure including the magnitude of maternal drug exposure and the relative distribution of drugs between fetal and maternal plasma (F_p_/M_p_) (35, 36). Differences between maternal and fetal plasma protein concentrations is a key factor impacting drug-specific F_p_/M_p_ values (36). By integrating maternal plasma protein concentration estimates from our analysis with data on fetal plasma protein concentrations, this work can contribute toward improving predictions of fetal drug exposure in pregnant individuals living with HIV.

Although many physiological changes transpire post-delivery, our current understanding of the time-course of such changes is limited. In studies evaluating ARV pharmacology in pregnant and postpartum individuals, postpartum individuals are typically evaluated at a single time point several weeks after delivery (i.e., ≥6 weeks) and merely serve as a control group to permit for assessments of pregnancy-associated changes in ARV PK (37–39). Use of PBPK modeling may offer an alternative approach for predicting the time-course of PK changes that occur immediately post-delivery and beyond. A recent publication by Dallmann et al. highlighted the potential utility of such models (17). The researchers developed a PBPK model for amoxicillin in pregnancy (3^rd^ trimester) and the early postpartum period (1.5–3.8 h post-delivery). The model was able to recapitulate observed PK data in postpartum individuals, albeit with a slight underprediction of clearance. Our findings will contribute to the development of such models for individuals living with HIV by improving our understanding of how plasma protein concentrations change during the postpartum period.

Our analysis distinctly differs from other previously published research describing the time-course of plasma protein concentrations in pregnant and postpartum individuals (7, 8, 17). Previously published equations were generated using aggregated data from literature sources that focused on Caucasian individuals with low-risk pregnancies and no other medical conditions (7, 8, 17). In contrast, our analysis utilized data from individuals living with HIV and included a racially diverse cohort. In terms of methodology, our analysis utilized a generalized additive modeling approach to fit non-parametric smoothers to the data. An advantage of this approach is that the form of the relationship between variables is driven by the dataset itself (40); whereas, for parametric models, which were used to develop the previously published equations, the form of the relationship between variables is pre-defined by the user (7, 8, 17). As previous studies describing the time-course of plasma protein concentrations in pregnant and postpartum individuals living with HIV are lacking, use of generalized additive modeling was considered to be additionally beneficial, permitting for trajectories to be derived based on the current dataset rather than relying on patterns depicted from other populations. Use of a generalized additive modeling approach also permitted us to evaluate the distributional form of albumin concentrations, which was possible due to the availability of individualized data from IMPAACT's P1026s study. In contrast, previously published equations were developed using aggregated data from the literature, making such distributional evaluations challenging (7, 8, 17). For development population PBPK models, there is an interest in generating virtual populations whose anatomy and physiology vary according to biologically relevant distributions. Mismatches in the distribution between virtually generated populations and the target population will serve to reduce confidence in model predictions. Our analysis demonstrated a high-level of incongruence between albumin quantiles generated from a previously published equation and observed data from pregnant individuals living with HIV, particularly at higher percentile values (Table 2) (8). Accordingly, use of the above-described equation to generate a virtual population reflective of pregnant individuals living with HIV would likely result in biased estimates of PK variability for drugs that are highly protein-bound. By characterizing the distributional form of serum albumin concentrations, our work can serve to improve confidence in population PBPK model predictions for pregnant and postpartum individuals living with HIV.

There are several limitations associated with our analysis that should be highlighted. First, a relatively low number of AAG samples were available for analysis. Estimated smoothing splines depicted linear relationships between AAG concentrations and gestational age/postpartum duration. As smoothing splines are fit using penalized regression, it is likely that the amount of observed data was insufficient to permit the model from deviating from a linear approximation owing to a relatively high (smoothness) penalty factor (41). Correspondingly, the presented work should be viewed as a preliminary analysis of the time-course of AAG concentrations in pregnant and postpartum individuals living with HIV. Second, for several individuals within each dataset, protein concentrations were measured longitudinally (i.e., contributing multiple samples). As our analysis approach (i.e., generalized linear modeling) implicitly assumed datapoints were independent, the presence of multiple observations per individuals may have inserted some bias into our model estimates. Nonetheless, as the majority of individuals contributed ≤ 2 samples (e.g., 270/380 and 230/354 participants contributed ≤ 2 albumin concentrations to the pregnancy and postpartum datasets, respectively) toward each respective dataset, development of a mixed-effect model to account for such features (i.e., multiple samples per patient) was not pursued. Lastly, although our analysis depicted unique trends in the time-course of protein concentrations for individuals living with HIV (Figures 2, 4), it lacked the capacity to identify the causative factor perpetuating such discrepancies. Other investigations have also observed lower albumin concentrations among cohorts of subjects with HIV-infection relative to non-HIV infected subjects (42, 43). However, due to demographic and socioeconomic differences that exist between individuals living with HIV and individuals without HIV infection, there may be several etiological factors responsible for such differences (e.g., racial, ethnic, nutritional, pathophysiological, etc.). As a preliminary analysis for this work, assessment of the trajectory of albumin concentrations by race was conducted (Supplementary Figure 3). This comparison did not provide striking evidence for racial differences in the time-course of albumin concentrations in pregnant and postpartum individuals living with HIV. Correspondingly, a generalized approach that described the time-course of protein concentrations across the entire cohort was adopted.

In summary, our analysis characterized the trajectory of serum albumin and plasma AAG concentrations in pregnant and postpartum individuals living with HIV. This work is particularly informative for the development of population PBPK models for this population, as their physiology may not be adequately described within current models. The results of our analysis have been compiled into computational functions developed using open-source software, permitting for easy integration into prospectively developed PBPK models. By doing so, our results can serve to inform the development of future clinical trials evaluating ARV pharmacology in pregnant and postpartum individuals living with HIV.

## Data Availability Statement

The data analyzed in this study is subject to the following licenses/restrictions: Due the ethical restrictions in the study's informed consent documents and in the International Maternal Pediatric Adolescent AIDS Clinical Trials (IMPAACT) Network's approved human subjects protection plan, data cannot be made publicly available. However, data can be made available to interested researchers upon request to the IMPAACT Statistical and Data Management Center's data access committee with the agreement of the IMPAACT Network. Requests to access these datasets should be directed to dac.data@fstrf.org.

## Ethics Statement

The studies involving human participants were reviewed and approved by all relevant Ethics or Human Subjects Protection Committees at all participating sites. IMPAACT P1026s is registered at ClinicalTrials.gov (https://clinicaltrials.gov/ct2/show/NCT00042289). All participants contributing data to the current analysis gave written informed consent, permission or assent in accordance with the Declaration of Helsinki. The patients/participants provided their written informed consent to participate in this study.

## Author Contributions

All co-authors reviewed, revised for content, and approved this article. SZ, MGo, JM, BB, MM, and AM made substantial contributions to the conception and design. BB and MM acquisition of data. SZ, MGo, MGr, HS, MM, KS, YB, JW, EC, BB, TJ, JM, and AM analysis or interpretation of data. All authors contributed to the article and approved the submitted version.

## Funding

Overall support for the International Maternal Pediatric Adolescent AIDS Clinical Trials Network (IMPAACT) was provided by the National Institute of Allergy and Infectious Diseases (NIAID) with co-funding from the Eunice Kennedy Shriver National Institute of Child Health and Human Development (NICHD) and the National Institute of Mental Health (NIMH), all components of the National Institutes of Health (NIH), under Award Numbers UM1AI068632 (IMPAACT LOC), UM1AI068616 (IMPAACT SDMC) and UM1AI106716 (IMPAACT LC), and by NICHD contract number HHSN275201800001I. This project was supported by an appointment to the Research Fellowship Program at the Office of New Drugs, Center for Drug Evaluation and Research, U.S. Food and Drug Administration, administered by the Oak Ridge Institute for Science and Education through an interagency agreement between the U.S. Department of Energy and FDA (SZ and MGo). AM receives research support from the non-profit organization, Thrasher Research Fund (www.thrasherresearch.org).

## Author Disclaimer

The content is solely the responsibility of the authors and does not necessarily represent the official views of the NIH. The opinions expressed in this article are those of the authors and should not be interpreted as the position of the U.S. FDA and the cases being presented do not imply FDA's endorsement of the products.

## Conflict of Interest

The authors declare that the research was conducted in the absence of any commercial or financial relationships that could be construed as a potential conflict of interest.

## Publisher's Note

All claims expressed in this article are solely those of the authors and do not necessarily represent those of their affiliated organizations, or those of the publisher, the editors and the reviewers. Any product that may be evaluated in this article, or claim that may be made by its manufacturer, is not guaranteed or endorsed by the publisher.

## Footnotes

^1^This article uses the term “pregnant individuals” to refer to all persons who can become pregnant regardless of gender identity. The terms “pregnant women living with HIV (PWLH)” and “postpartum women” are used in the Methods and Results section of the article because IMPAACT 1026s protocol specified that women were to be enrolled.
