# Supplementary material for: Characterization of Plasma Protein Alterations in Pregnant and Postpartum Individuals Living With HIV to Support Physiologically-Based Pharmacokinetic Model Development
Source: Front Pediatr. 2021 Oct 13;9:721059. doi: 10.3389/fped.2021.721059 (PMC8550258; doi:10.3389/fped.2021.721059)
Supplement: Supplementary file 2 [file Data_Sheet_2.docx]

**Supplemental Material**

**Manuscript title:** Characterization of Plasma Protein Alterations in Pregnant and Postpartum Individuals Living with HIV to Support Physiologically-based Pharmacokinetic Model Development

**Table of Contents**

Page 1: Table of Contents

Page 2: Supplementary Table S1

Page 3: Supplementary Table S2

Page 4: Supplementary Table S3

Page 5: Supplementary Table S4

Page 6: Supplementary Table S5

Page 7: Supplementary Figure S1

Page 8: Supplementary Figure S2

Page 9: Supplementary Figure S3

Page 10: Supplementary R functions

Page 15: Supplementary Example R script

Page 18: Supplementary References

**Supplementary Table S1**: Concomitant antiretrovirals for women enrolled in IMPAACT’s P1026s who contributed serum albumin and plasma α1-acid glycoprotein concentrations towards the analysis

| Drug class  Antiretroviral, n (%)^b^ | Albumin Pregnancy Dataset  N = 380^a^ | Albumin Postpartum Dataset  N = 354^a^ | AAG Pregnancy Dataset  N = 31^a^ | AAG Postpartum Dataset  N = 30^a^ |
| --- | --- | --- | --- | --- |
| Entry Inhibitor  maraviroc | 3 (0.8%) | 2 (0.6%) | - | - |
| INSTI  elvitegravir  dolutegravir  raltegravir | 15 (3.9%)  24 (6.3%)  48 (12.6%) | 14 (4%)  19 (5.4%)  45 (12.7%) | -  -  - | -  -  - |
| Fusion Inhibitor  enfuvirtide | 2 (0.5%) | 2 (0.6%) | 1 (3.2%) | - |
| NRTI  abacavir  didanosine  emtricitabine  lamivudine  stavudine  tenofovir  zidovudine | 67 (17.6%)  10 (2.6%)  161 (42.4%)  216 (56.8%)  13 (3.4%)  203 (53.4%)  298 (78.4%) | 61 (17.2%)  10 (2.8%)  152 (42.9%)  196 (55.4%)  12 (3.4%)  192 (54.2%)  277 (78.2%) | 8 (25.8%)  3 (9.7%)  3 (9.7%)  25 (80.6%)  2 (6.5%)  7 (22.6%)  31 (100%) | 7 (23.3%)  2 (6.7%)  2 (6.7%)  24 (80%)  1 (3.3%)  6 (20%)  30 (100%) |
| NNRTI  efavirenz  etravirine  nevirapine  rilpivirine | 26 (6.8%)  4 (1.1%)  13 (3.4%)  1 (0.3%) | 24 (6.8%)  3 (0.8%)  11 (3.1%)  1 (0.3%) | 3 (9.7%)  -  1 (3.2%)  - | 3 (10%)  -  -  - |
| PI  atazanavir  darunavir  fosamprenavir  lopinavir  nelfinavir  saquinavir | 118 (31.1%)  73 (19.2%)  5 (1.3%)  110 (28.9%)  52 (13.7%)  3 (0.8%) | 111 (31.4%)  70 (19.8%)  4 (1.1%)  102 (28.8%)  50 (14.1%)  3 (0.8%) | 2 (6.5%)  -  -  31 (100%)  1 (3.2%)  - | 2 (6.7%)  -  -  30 (100%)  1 (3.3%)  - |
| Booster  cobicistat  ritonavir | 15 (3.9%)  286 (75.3%) | 14 (4%)  268 (75.7%) | -  31 (100%) | -  30 (100%) |

AAG: alpha 1-acid glycoprotein; INSTI: integrase strand transfer inhibitor; NRTI: nucleoside reverse transcriptase inhibitor; NNRTI: non-nucleoside reverse transcriptase inhibitor; and PI: protease inhibitor

^a^N = Number of participants included in each dataset. As participants received multiple medications, the sum participants receiving each medication will exceed the total number of participants in each each dataset.

^b^n(%) = number (percent) of participants receiving the denoted medication

**Supplementary Table S2**: Comparison of Akaike Information Criterion values for models estimating serum albumin in pregnant women living with HIV (PWLH)

| **Error Distributions^b^** | **Akaike Information Criterion (AIC)^a^** | |
| --- | --- | --- |
|  | Models accounting for time-dependent changes in the central tendency (location) of albumin concentrations^c^ | Models accounting for time-dependent changes in the central tendency (location) AND variance (scale) of albumin concentrations^d^ |
| Normal mixture^e^ | **4659.450** | 4657.416 |
| Skew normal type 2 | 4689.794 | - |
| Weibull | 4698.555 | - |
| Generalised gamma | 4700.543 | - |
| Box-Cox Cole-Green | 4712.206 | - |
| Gumbel | 4715.302 | - |
| Normal | 4755.987 | - |
| Skew normal type 1 | 4757.880 | - |
| t Family | 4757.988 | - |
| Normal family | 4758.439 | - |
| Gamma | 4797.584 | - |
| Log normal | 4822.850 | - |
| Inverse Gaussian | 4824.141 | - |
| Generalised inv. Gaussian | ERR^f^ | - |
| Exponential Gaussian | ERR^f^ | - |

Selected model AIC value is bolded

^a^Generalized Akaike Information Criterion computed as -2*LL + p*df, where LL is the fitted log-likelihood, p is a penalty factor of 2, and df is the total degrees of freedom of the model.

^b^Additional details for evaluated error distributions are provided in (1)

^c^Models parameterized using penalized beta splines to describe the relationship between the central tendency of serum albumin concentrations (response variable) and gestational age (explanatory variable). All models were developed using a similar functional form: Albumin ~ Intercept + pb(GAW), where pb(GAW) describes a penalized beta spline that is a function of gestational age.

^d^Model parameterized using separate penalized beta splines to describe the relationship between the central tendency and variance of serum albumin concentrations (response variable) with increasing gestational age (explanatory variable). Only the best performing error distribution model, based on evaluations of the AIC (i.e., lowest value) for models accounting for changes in the central tendency of albumin concentrations alone, was the inclusion of time-dependent changes in variance evaluated.

^e^Models fit using the gamlss() function in R with the exception of models accommodating mixture-normal distributions, which were fit using the gamlssNP() function.

^f^Computational error (i.e., estimation failed)

**Supplementary Table S3**: Serum albumin concentrations (871 samples) in 380 PWLH

| Weeks of Gestation | Number of Samples | Observed Serum Albumin, g/L^a^ | Predicted Serum Albumin, g/L^b^ |
| --- | --- | --- | --- |
| <20^c^ | 2 | 36 (2.83) | - |
| 20 – <22 | 29 | 34.34 (3.88) | 34.36 |
| 22– <24 | 51 | 34.84 (3.49) | 34.36 |
| 24 – <26 | 58 | 34.21 (3.61) | 34.22 |
| 26 – <28 | 40 | 33.58 (3.61) | 33.97 |
| 28 – <30 | 56 | 33.63 (3.95) | 33.65 |
| 30 – <32 | 119 | 33.6 (3.46) | 33.35 |
| 32 – <34 | 166 | 33.15 (3.67) | 33.16 |
| 34 – <36 | 191 | 33.1 (3.82) | 33.16 |
| 36 – <38 | 116 | 32.9 (3.79) | 33.41 |
| 38 – <40 | 38 | 34.43 (3.87) | 33.93 |
| 40 – <42 | 4 | 36.73 (2.26) | 34.67 |
| 42 – <44 | 1 | 36 (NA) | 35.5 |

NA, not applicable

^a^Arithmetic mean (SD)

^b^Model estimated (arithmetic) mean serum albumin concentration. Estimates are reflective of the middle of each gestational age range (e.g., for 20 **–** <22 weeks, an estimate corresponding to 21 weeks gestation was reported). An estimate for <20 weeks gestation was not generated.

^c^Serum albumin concentrations were collected from two participants at 19.7 and 19.9 weeks gestation. These time-points were considered to be ~20 weeks gestation and were not inferred to represent a violation of the protocol’s inclusion criteria (i.e., ≥20 weeks gestation).

**Supplementary Table S4**: Comparison of Akaike Information Criterion values for models estimating serum albumin in postpartum women living with HIV

| **Error Distributions^b^** | **Akaike Information Criterion (AIC)^a^** | |
| --- | --- | --- |
|  | Models accounting for time-dependent changes in the central tendency (location) of albumin concentrations^c^ | Models accounting for time-dependent changes in the central tendency (location) AND variance (scale) of albumin concentrations^d^ |
| Skew normal type 2 | 4230.999 | 4207.804 / **4206.738**^#^ |
| Normal mixture^e^ | 4247.636 | - |
| t Family | 4254.192 | - |
| Normal | 4265.292 | - |
| Skew normal type 1 | 4265.487 | - |
| Gumbel | 4268.956 | - |
| Box-Cox Cole-Green | 4277.857 | - |
| Weibull | 4281.621 | - |
| Generalised gamma | 4283.024 | - |
| Normal family | 4284.270 | - |
| Gamma | 4375.638 | - |
| Log normal | 4406.389 | - |
| Inverse Gaussian | 4459.285 | - |
| Generalised inv. Gaussian | ERR^f^ | - |
| Exponential Gaussian | ERR^f^ | - |

Selected model AIC value is bolded

^a^Generalized Akaike Information Criterion computed as -2*LL + p*df, where LL is the fitted log-likelihood, p is a penalty factor of 2, and df is the total degrees of freedom of the model.

^b^Additional details for evaluated error distributions are provided in (1)

^c^Models parameterized using penalized beta splines to describe the relationship between the central tendency of serum albumin concentrations (response variable) and postpartum duration (explanatory variable). All models were developed using a similar functional form: Albumin ~ Intercept + pb(POSTP), where pb(POSTP) describes a penalized beta spline that is a function of postpartum duration (weeks).

^d^Model parameterized using separate penalized beta splines to describe the relationship between the central tendency and variance of serum albumin concentrations (response variable) with increasing postpartum duration (explanatory variable). Only the best performing error distribution model, based on evaluations of the AIC (i.e., lowest value) for models accounting for changes in the central tendency of albumin concentrations alone, was the inclusion of time-dependent changes in variance evaluated.

^e^Models fit using the gamlss() function in R with the exception of models accommodating normal mixture distributions, which were fit using the gamlssNP() function.

^f^Computational error (i.e., estimation failed)

^#^The initially evaluated model that included separate penalized beta splines to describe the relationship between the central tendency and variance of serum albumin concentrations (response variable) with increasing postpartum duration (explanatory variable) failed to converge after 100 iterations (AIC = 4207.804). An alternative model that permitted for separate variance estimates for albumin concentrations ≤0.5 weeks and >0.5 weeks postpartum converged and exhibited an AIC of 4206.738.

S**upplementary Table S5**: Serum albumin concentrations (757 samples) in 354 postpartum women living with HIV

| Weeks Postpartum | Number of Samples | Observed Serum Albumin, g/L^a^ | Predicted Serum Albumin, g/L^b^ |
| --- | --- | --- | --- |
| 0 – <2 | 231 | 31.97 (4.92) | 34.76^c^ |
| 2 – <4 | 153 | 38.3 (3.5) | 38.25 |
| 4 – <6 | 45 | 39.16 (3.44) | 39.73 |
| 6 – <8 | 110 | 40.25 (3.29) | 40.45 |
| 8 – <10 | 40 | 40.35 (4.83) | 40.98 |
| 10 – <12 | 50 | 42.38 (4.03) | 41.5 |
| 12 – <14 | 11 | 40.18 (5.19) | 41.99 |
| 14 – <16 | 5 | 42 (3.08) | 42.37 |
| 16 – <18 | 3 | 43 (2.65) | 42.57 |
| 18 – <20 | 3 | 42.33 (4.93) | 42.58 |
| 20 – <22 | 9 | 42.67 (3.35) | 42.45 |
| 22 – <24 | 8 | 43.62 (2.26) | 42.26 |
| 24 – <26 | 29 | 41.89 (4.14) | 42.13 |
| 26 – <28 | 34 | 41.91 (3.1) | 42.09 |
| 28 – <30 | 8 | 43.38 (3.85) | 42.17 |
| 30 – <32 | 8 | 42.25 (3.06) | 42.3 |
| 32– <34 | 7 | 42.14 (3.8) | 42.38 |
| ≥34 | 3 | 41.33 (3.51) | - |

NA, not applicable

^a^Arithmetic mean (SD)

^b^Model estimated (arithmetic) mean serum albumin concentration. Estimates are reflective of the middle of each postpartum duration range (e.g., for 0 **–** <2 weeks, an estimate corresponding to 1 week postpartum was reported). An estimate for ≥34 weeks postpartum was not generated.

^c^The majority of observed albumin samples collected during the first postpartum interval (0-<2 weeks) were attained on the day of delivery. This is demonstrated by evaluating the median postpartum duration for samples within this interval, 0 weeks. Of note, the model estimated (arithmetic) mean serum albumin concentration on the day of delivery (i.e., 0 weeks postpartum), 31.60 g/L, demonstrates an appropriate fit to the observed albumin concentrations during this time period.

**Supplementary Figure S1**: Quantile-quantile (Q-Q) plot of residuals for the selected model for serum albumin concentrations in PWLH. Sample quantiles depict normalized (randomized) quantile residuals.


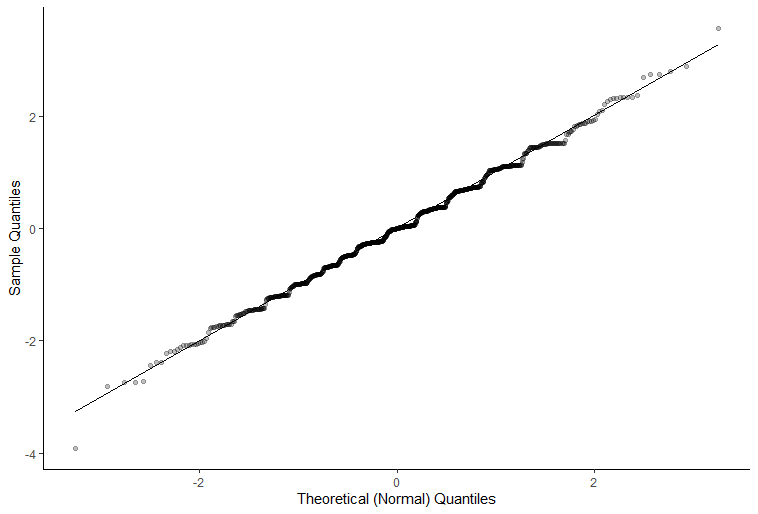


**Supplementary Figure S2**: Quantile-quantile (Q-Q) plot of residuals for the selected model for serum albumin concentrations in postpartum women living with HIV. Sample quantiles depict normalized (randomized) quantile residuals.


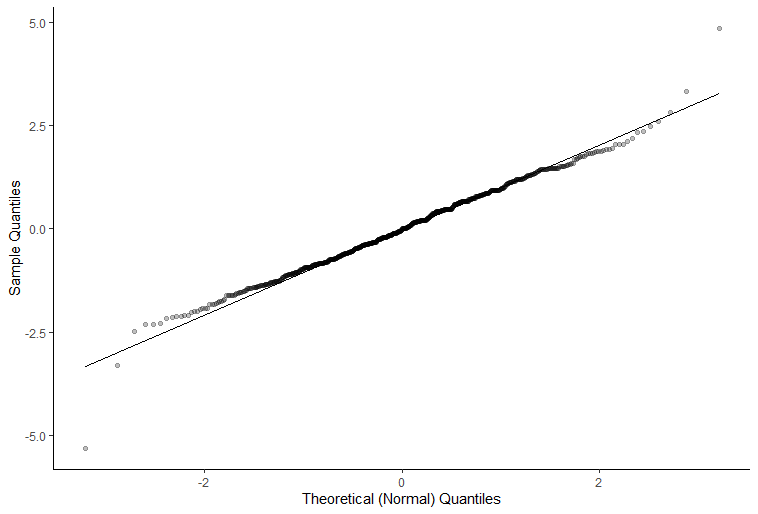


**Supplementary Figure S3**: Comparison of the time-course of serum albumin concentrations by race in (A) pregnant and (B) postpartum individuals living with HIV. Evaluated racial groups included (1) White, (2) Black (African and African American), and (3) Other. Separate generalized additive models corresponding to each racial group were fit to data from pregnant and postpartum individuals. Models were developed using smoothing splines to describe time-dependent changes in albumin concentrations and gaussian probability distributions. Lines depict model predictions. Circles denoted observed serum albumin concentrations color-coded correspondingly to the race of the individual.

**
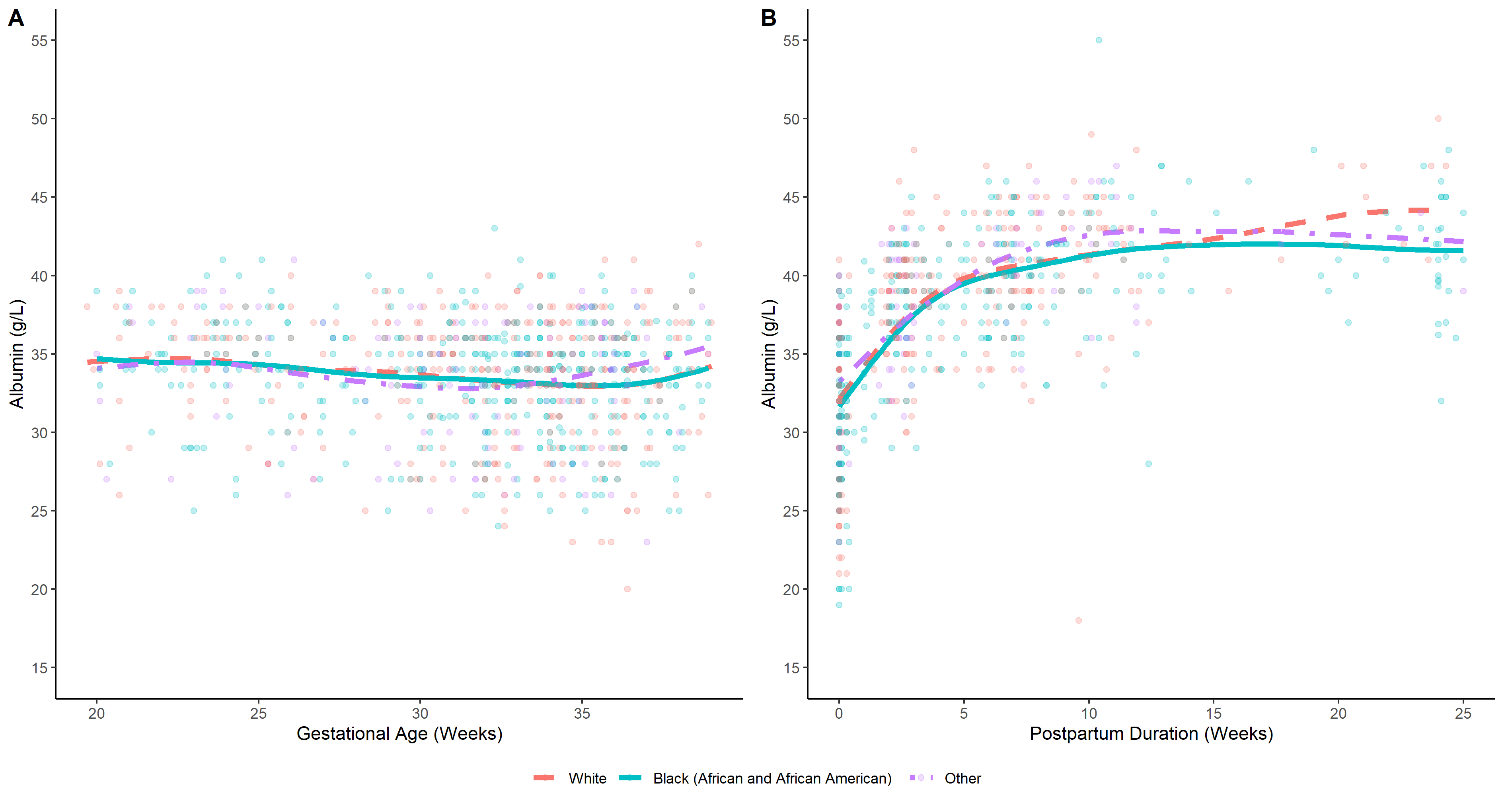
**

**Supplementary R functions**

*Function describing the arithmetic mean of serum albumin concentrations in PWLH*

alb_preg_mean<- function(GAW){

#input

#-GAW: An array of gestational age values (weeks) for pregnant women.

#output

#-An array of arithmetic mean serum albumin concentration values (g/L) corresponding to the input GAW values

#note

#-the dataset was developed using serum albumin values collected between 19.7 and 42.30 weeks GAW.

#predictions should only be generated for this range of GAW.

coef=c(1.008952e+02, -1.335985e+01, 1.028753e+00, -3.783570e-02, 6.619597e-04, -4.407826e-06)

return(coef[1]+(coef[2]*GAW^1)+(coef[3]*GAW^2)+(coef[4]*GAW^3)+(coef[5]*GAW^4)+(coef[6]*GAW^5))

}

*Function describing quantiles of serum albumin concentrations in PWLH*

alb_preg_quant<-function(p, GAW){

#input

#-p: An array of percentiles (range: >0 to <1) for which serum albumin values will be generated

#-GAW: An array of gestational age values (weeks) corresponding to pregnant women for which serum albumin values will be generated

##-the array sizes of 'p' and 'GAW' should be congruent (i.e., the same)

#output

#-An array of serum albumin concentration (g/L) quantiles corresponding to the 'p' and 'GAW'input arrays.

#-Estimates are indexed according to the input 'p' and 'GAW' values. For example, the 1st element of the output array corresponds

#to 1st elements of the input 'p' and 'GAW' arrays. The 2nd element of the output array corresponds to 2nd elements of the input

#'p' and 'GAW' arrays and so forth.

#note

#-the dataset was developed using serum albumin values collected between 19.7 and 42.30 weeks GAW.

#predictions should only be generated for this range of GAW.

#-this function requires the EnvStats package to be installed

if (require(EnvStats)==FALSE){

install.packages("EnvStats", dep = TRUE)

}

library(EnvStats)

polyPREG<- function(X, coef){return(coef[1]+(coef[2]*X^1)+(coef[3]*X^2)+(coef[4]*X^3)+(coef[5]*X^4)+(coef[6]*X^5))}

coef1=c(1.008952e+02, -1.335985e+01, 1.028753e+00, -3.783570e-02, 6.619597e-04, -4.407826e-06)

mean<-polyPREG(X = GAW, coef = coef1)

sd=2.318221

p.mix=0.7527338

mean1=mean-p.mix*(6.65007)

mean2=mean1+6.65007

output=NULL

for (i in 1:length(mean1)){

output=rbind(output, qnormMix(p = p[i], mean1 = mean1[i], sd1 = sd, mean2 = mean2[i], sd2 = sd, p.mix = p.mix))

}

return(as.numeric(output))

}

*Function describing the arithmetic mean of serum albumin concentrations in postpartum women living with HIV*

alb_postp_mean<- function(POSTPW){

#input

#-POSTPW: An array of postpartum duration values (weeks) for postpartum women.

#output

#-An array of arithmetic mean serum albumin concentration values (g/L) corresponding to the input POSTPW values

#note

#-the dataset was developed using serum albumin values predominately collected between 0 (i.e., day of birth) and 35.3 weeks POSTPW.

#predictions should only be generated for this range of POSTPW

coef=c(3.160331e+01, 3.816472e+00, -7.280251e-01, 7.735136e-02, -4.548616e-03, 1.468655e-04, -2.437508e-06, 1.624886e-08)

return(coef[1]+(coef[2]*POSTPW^1)+(coef[3]*POSTPW^2)+(coef[4]*POSTPW^3)+(coef[5]*POSTPW^4)+(coef[6]*POSTPW^5)+(coef[7]*POSTPW^6)+(coef[8]*POSTPW^7))

}

*Function describing quantiles of serum albumin concentrations in postpartum women living with HIV*

alb_postp_quant<-function(p, POSTPW){

#input

#-p: An array of percentiles (range: >0 to <1) for which serum albumin values will be generated

#-POSTPW: An array of postpartum duration values (weeks) corresponding to postpartum women for which serum albumin values will be generated

##-the array sizes of 'p' and 'POSTPW' should be congruent (i.e., the same)

#output

#-An array of serum albumin concentration (g/L) quantiles corresponding to the 'p' and 'POSTPW' input arrays.

#-Estimates are indexed according to the input 'p' and 'POSTPW' values. For example, the 1st element of the output array corresponds

#to 1st elements of the input 'p' and 'POSTPW' arrays. The 2nd element of the output array corresponds to 2nd elements of the input

#'p' and 'POSTPW' arrays and so forth.

#note

#-the dataset was developed using serum albumin values predominately collected between 0 (i.e., day of birth) and 35.3 weeks POSTPW.

#predictions should only be generated for this range of POSTPW

polyPOSTP<- function(X, coef){return(coef[1]+(coef[2]*X^1)+(coef[3]*X^2)+(coef[4]*X^3)+(coef[5]*X^4)+(coef[6]*X^5)+(coef[7]*X^6)+(coef[8]*X^7))}

coef1=c(3.160331e+01, 3.816472e+00, -7.280251e-01, 7.735136e-02, -4.548616e-03, 1.468655e-04, -2.437508e-06, 1.624886e-08)

mean<-polyPOSTP(X = POSTPW, coef = coef1)

sd=ifelse(POSTPW<=0.5, 4.500003, 3.367181)

nu=0.7179217

mode=mean-sd*(sqrt(2)/sqrt(pi))*(nu-(nu^-1))

output=NULL

for (i in 1:length(p)){

if(p[i] <=((1+nu^2)^-1)){

output=rbind(output, mode[i]+(sd[i]/nu)*qnorm(p[i]*(1+nu^2)/2) )

}else{

output=rbind(output, mode[i]+(sd[i]*nu)*qnorm( (p[i]*(1+nu^2)-1+nu^2)/(2*nu^2)) )

}#endif

}#end loop

return(as.numeric(output))

}

*Function describing the arithmetic mean of plasma AAG concentrations in PWLH*

aag_preg_mean<- function(GAW){

#input

#-GAW: An array of gestational age values (weeks) for pregnant women.

#output

#-An array of arithmetic mean plasma alpha1-acid glycoprotein concentration values (mg/dL) corresponding to the input GAW values

#note

#-the dataset was developed using plasma alpha1-acid glycoprotein values collected between 23.4 and 37.9 weeks GAW.

#predictions should only be generated for this range of GAW.

coef=c(69.713597, -0.671988)

return(coef[1]+(coef[2]*GAW^1))

}

*Function describing the arithmetic mean of plasma AAG concentrations in postpartum women living with HIV*

aag_postp_mean<- function(POSTPW){

#input

#-POSTPW: An array of postpartum duration values (weeks) for postpartum women.

#output

#-An array of arithmetic mean plasma alpha1-acid glycoprotein concentration values (mg/dL) corresponding to the input POSTPW values

#note

#-the dataset was developed using plasma alpha1-acid glycoprotein values collected between 1.7 and 12.9 weeks POSTPW.

#predictions should only be generated for this range of POSTPW

coef=c(129.580535, -4.570736)

return(coef[1]+(coef[2]*POSTPW^1))

}

**Supplementary Example R Script**

##########ALBUMIN FUNCTIONS#######################

######################################################

######PREGNANCY FUNCTION EXAMPLES#################

##pregnancy functions should only be used to estimate serum albumin levels within the range of data used for equation development: 19.7 and 42.30 weeks gestation

###1. estimate arithmetic mean serum albumin values (g/L) for PWLH at 20, 28, and 37 weeks gestation

alb_preg_mean(GAW = 20) #34.32231

alb_preg_mean(GAW = 28) #33.80966

alb_preg_mean(GAW = 37) #33.41485

#or

alb_preg_mean(GAW=c(20,28,37)) #34.32231 33.80966 33.41485

###2. estimate serum albumin median values (g/L) for PWLH at 20, 28, and 37 weeks gestation

alb_preg_quant(p = 0.5, GAW = 20) #34.9984

alb_preg_quant(p = 0.5, GAW = 28) #34.48575

alb_preg_quant(p = 0.5, GAW = 37) #34.09094

#or

alb_preg_quant(p=c(0.5,0.5,0.5), GAW=c(20,28,37)) #34.99840 34.48575 34.09094

##3. estimate serum albumin quantile values (g/L) for PWLH corresponding to

#the the 5th %tile at 20 weeks; 85th %tile at 28 weeks; and the 65th %tile at 37 weeks gestation.

alb_preg_quant(p = 0.05, GAW = 20) #27.3811

alb_preg_quant(p = 0.85, GAW = 28) #37.41136

alb_preg_quant(p = 0.65, GAW = 37) #35.26597

#or

alb_preg_quant(p=c(0.05,0.85,0.65), GAW=c(20,28,37)) #27.38110 37.41136 35.26597

##4. population generation - produce serum albumin concentration values (g/L) for a population of 100 PWLH at 20 weeks gestation

nx=100

rand=runif(n=nx) #creates random percentile values between 0 and 1.

out=alb_preg_quant(p=rand, GAW=rep(20, nx))

print(out)

hist(out, breaks=20 ,main = 'Histogram of Serum Albumin Values @ 20 weeks gestation', xlab='Albumin (g/L)' )

########POSTPARTUM FUNCTION EXAMPLES#################

## postpartum functions should only be used to estimate serum albumin levels within the range 0 (i.e., day of birth) to 35.3 weeks postpartum, which represents the collection interval that the majority of observed data was collected over

###1. estimate arithmetic mean serum albumin values (g/L) for postpartum women living with HIV at 0 (on day of birth), 15, and 30 weeks postpartum

alb_postp_mean(POSTPW = 0) #31.60331

alb_postp_mean(POSTPW = 15) #42.36942

alb_postp_mean(POSTPW = 30) #42.23353

#or

alb_postp_mean(POSTPW = c(0,15,30)) #31.60331 42.36942 42.23353

###2. estimate serum albumin median values (g/L) for postpartum women living with HIV at 0 (on day of birth), 15, and 30 weeks postpartum

alb_postp_quant(p = 0.5, POSTPW = 0) #32.09317

alb_postp_quant(p = 0.5, POSTPW = 15) #42.73596

alb_postp_quant(p = 0.5, POSTPW = 30) #42.60007

#or

alb_postp_quant(p = c(0.5,0.5,0.5), POSTPW = c(0,15,30)) #32.09317 42.73596 42.60007

##3. estimate serum albumin quantile values (g/L) for postpartum women living with HIV corresponding to

#the the 5th %tile at 0 weeks; 85th %tile at 15 weeks; and the 65th %tile at 30 weeks postpartum.

alb_postp_quant(p = 0.05, POSTPW = 0) #22.89613

alb_postp_quant(p = 0.85, POSTPW = 15) #46.04533

alb_postp_quant(p = 0.65, POSTPW = 30) #43.95889

#or

alb_postp_quant(p = c(0.05,0.85,0.65), POSTPW = c(0,15,30)) #22.89613 46.04533 43.95889

##4. population generation - produce serum albumin concentration values (g/L) for a population of 100 postpartum women living with HIV at 15 weeks postpartum

nx=100

rand=runif(n=nx) #creates random percentile values between 0 and 1.

out=alb_postp_quant(p=rand, POSTPW = rep(15, nx))

print(out)

hist(out, breaks=20 ,main = 'Histogram of Serum Albumin Values @ 15 weeks postpartum', xlab='Albumin (g/L)' )

##########ALPHA1-ACID GLYCOPROTEIN FUNCTIONS######

#########################################################

######PREGNANCY FUNCTION EXAMPLE#################

## pregnancy functions should only be used to estimate plasma alpha1-acid glycoprotein levels within the range of data used for equation development: 23.4 and 37.9 weeks gestation

###1. estimate arithmetic mean plasma alpha1-acid glycoprotein values (mg/dL) for PWLH at 20, 28, and 37 weeks gestation

aag_preg_mean(GAW = 20) #56.27384

aag_preg_mean(GAW = 28) #50.89793

aag_preg_mean(GAW = 37) #44.85004

#or

aag_preg_mean(GAW=c(20,28,37)) #56.27384 50.89793 44.85004

########POSTPARTUM FUNCTION EXAMPLE#################

##postpartum functions should only be used to estimate plasma alpha1-acid glycoprotein levels within the range of data used for equation development: 1.7 to 12.9 weeks postpartum

###1. estimate arithmetic mean plasma alpha1-acid glycoprotein values (mg/dL) for postpartum women living with HIV at 2, 7, and 12 weeks postpartum

aag_postp_mean(POSTPW = 2) #120.4391

aag_postp_mean(POSTPW = 7) #97.58538

aag_postp_mean(POSTPW = 12) #74.7317

#or

aag_postp_mean(POSTPW = c(2,7,12)) #120.43906 97.58538 74.73170

**Supplementary References**

1. Rigby RA, Heller GZ, Stasinopoulos MD, De Bastiani F. Distributions for modelling location, scale, and shape: using GAMLSS in R. CRC Press; 2017.
